# Abnormal functional connectivity under somatosensory stimulation in migraine: a multi-frequency magnetoencephalography study

**DOI:** 10.1186/s10194-019-0958-3

**Published:** 2019-01-09

**Authors:** Jing Ren, Jing Xiang, Yueqiu Chen, Feng Li, Ting Wu, Jingping Shi

**Affiliations:** 1 Department of Neurology, The Affiliated Brain Hospital of Nanjing Medical University, Nanjing, 210029 Jiangsu China; 2 MEG Center, Division of Neurology, Cincinnati Children’s Hospital Medical Center, Cincinnati, OH 45220 USA; 3 MEG Center, Nanjing Brain Hospital, Nanjing, 210029 Jiangsu China

**Keywords:** Migraine, Magnetoencephalography, Multi-frequency, Somatosensory, Functional connectivity

## Abstract

**Background:**

Although altered neural networks have been demonstrated in recent MEG (magnetoencephalography) research in migraine patients during resting state, it is unknown whether this alteration can be detected in task-related networks. The present study aimed to investigate the abnormalities of the frequency-specific somatosensory-related network in migraine patients by using MEG.

**Methods:**

Twenty-two migraineurs in the interictal phase and twenty-two sex- and age-matched healthy volunteers were studied using a whole-head magnetoencephalography (MEG) system. Electrical stimuli were delivered alternately to the median nerve on the right wrists of all subjects. MEG data were analyzed in a frequency range of 1–1000 Hz in multiple bands.

**Results:**

The brain network patterns revealed that the patients with migraine exhibited remarkably increased functional connectivity in the high-frequency (250–1000 Hz) band between the sensory cortex and the frontal lobe. The results of quantitative analysis of graph theory showed that the patients had (1) an increased degree of connectivity in the theta (4–8 Hz), beta (13–30 Hz) and gamma (30–80 Hz) bands; (2) an increased connectivity strength in the beta (13–30 Hz) and gamma (30–80 Hz) bands; (3) an increased path length in the beta (13–30 Hz), gamma (30–80 Hz) and ripple (80–250 Hz) bands; and (4) an increased clustering coefficient in the theta (4–8 Hz), beta (13–30 Hz) and gamma (30–80 Hz) bands.

**Conclusions:**

The results indicate that migraine is associated with aberrant connections from the somatosensory cortex to the frontal lobe. The frequency-specific increases in connectivity in terms of strength, path length and clustering coefficients support the notion that migraineurs have elevated cortical networks. This alteration in functional connectivity may be involved in somatosensory processing in migraine patients and may contribute to understanding migraine pathophysiology and to providing convincing evidence for a spatially targeted migraine therapy.

## Introduction

Migraine is a common neurological disorder accompanied by nausea, vomiting, yawning, photophobia, and phonophobia [1]. The pathogenesis of migraine remains unclear. Recent analyses tend to define migraine as a brain dysfunction disease rather than a blood-vessel disorder [2, 3]. In a previous report [4], the human brain was described as a complex network of several different functional brain regions that constantly share information with each other. Functional communication between separated brain regions is of great importance in complex brain processes, and it thrives in the continuous organization of information among different parts of the brain [4, 5]. Thus, the functional connectivity of the human brain is critical to its integration. Functional connectivity has been used to investigate brain function, and it has confirmed the altered functional network in migraine patients in a resting state [6–8]. Graph theory has been proposed and developed by scientists to quantify varying networks and describe the properties of the brain network distribution [9].

Cortical activation induced by external stimuli has drawn attention in past years [10–12]. The study of connectivity in regard to synchronization and information processing has revealed differences under visual stimulation between migraineurs and controls [13, 14]; this result suggested that these methods would be useful for outlining stimulus processing in migraine and providing further information on how the internal regions change their connections under the influence of external inputs. An electroencephalogram (EEG) study [15] has demonstrated abnormal functional connectivity in EEG signals in laser reactivity in migraineurs. Similar to other types of tasks, several studies have discovered that migraine patients during and between migraine attacks have altered stimuli-induced activation. The alternation can occur in any brain area that participates in sensory processing such as the cortical and subcortical regions and the brainstem [16]. Functional brain-imaging studies in migraine have verified the existence of atypical stimulus-induced activations and abnormal functional networks among brain areas that participate in sensory processing [17].

Magnetoencephalography (MEG) is a noninvasive technology for studying the function of the central nervous system in a wide frequency range. Compared to previous neuroimaging methods, such as functional magnetic resonance imaging (fMRI) and EEG, MEG shows remarkable spatiotemporal resolution, and it can reveal subtle differences in brain activity [18]. Although various artifacts including muscle, eye movement, and filtering present a challenge in high-frequency oscillations (HFOs) analysis, high-frequency evaluation in MEG still has the advantage of high sensor density, which covers the whole head [19]. Using the state-of-the-art MEG technology, somatosensory-evoked response following electrical median-nerve stimulation has revealed neuromagnetic signals from the conventional low (< 100 Hz) to very high (approximately 1000 Hz) frequency ranges [20–22]. However, it remains unclear whether frequency-specific neural networks play a role in migraine.

The objective of the present study was to investigate the somatosensory-related functional networks in migraine patients. Our central hypothesis is that the pattern and topology of functional connectivity under sensory stimuli in patients with migraine is significantly altered compared with that in controls. To our knowledge, to date, the current study is the first to apply functional connectivity for neural network analysis under somatosensory stimulus in such a wide frequency range (1–1000 Hz) to detect differences in sensory information transfer between migraine patients and healthy volunteers.

## Methods

### Subjects

Twenty-two migraine patients without aura and whose dominant hand is right were chosen from Nanjing Brain Hospital. The diagnosis criteria of migraine comply with the International Classification of Headache Disorders, 3rd edition (ICHD-IIIbeta) of 2013 (Headache Classification Subcommittee of the International Headache Society, 2013) [23]. Exclusion criteria for migraineurs included the presence of any other neurological disorders. The healthy participants never reported any history of migraine or occurrence of other types of headache. All subjects with a ferromagnetic implant, a history of brain damage, an inability to stay still, and use of the drug within 1 month before the test were excluded (except for preventative medicine or acute medication in migraine sufferers). The medical ethics committee of Nanjing Brain Hospital approved the research protocol, and each subject provided written informed consent.

### Sensory stimuli

All participants were required to experience a 0.2 ms duration electrical stimuli delivered to the median nerve by turns at their right wrists. The stimulation intensity was just greater than the motor threshold (thumb movement) and was never reported as painful. During somatosensory-evoked magnetic fields (SEFs) samplings, the subjects were instructed to close their eyes and relax their muscles without focusing on the external stimuli.

### MEG recordings

The MEG recordings were obtained in a magnetically shielded room using a whole-head CTF 275-channel MEG system (VSM Medical Technology Company, Canada). The recording required that no subject should have experienced headache and pain seizures for at least 72 h prior to testing or migraine precipitation during or after sampling. During the data sampling, each subject was asked to lie comfortably in a positive supine, rest their limbs, close their eyes and avoid moving their head or swallowing or clenching their teeth during the entire process. Before initiating data acquisition, three electromagnetic coils were attached to reference landmarks on the left and right pre-auricular points and the nasion of each participant to check the head position. Head position moves exceeding approximately 1.5 cm were excluded. The sampling rate of MEG recording was 6000 Hz. Noise cancellation in the recorded data was performed continuously online with third-order gradients.

### MRI scan

Structural magnetic resonance imaging (MRI) of all participants were scanned using a 1.5-T MRI (Singa, GE, USA). We placed three fiducial markers in locations identical to the positions of the three coils used during the MEG data acquisition to facilitate co-registration of the two data sets. Subsequently, all anatomical landmarks digitized in the MEG study were identified on MRI.

### Data analysis

#### Morphology

**Fig. 1:**
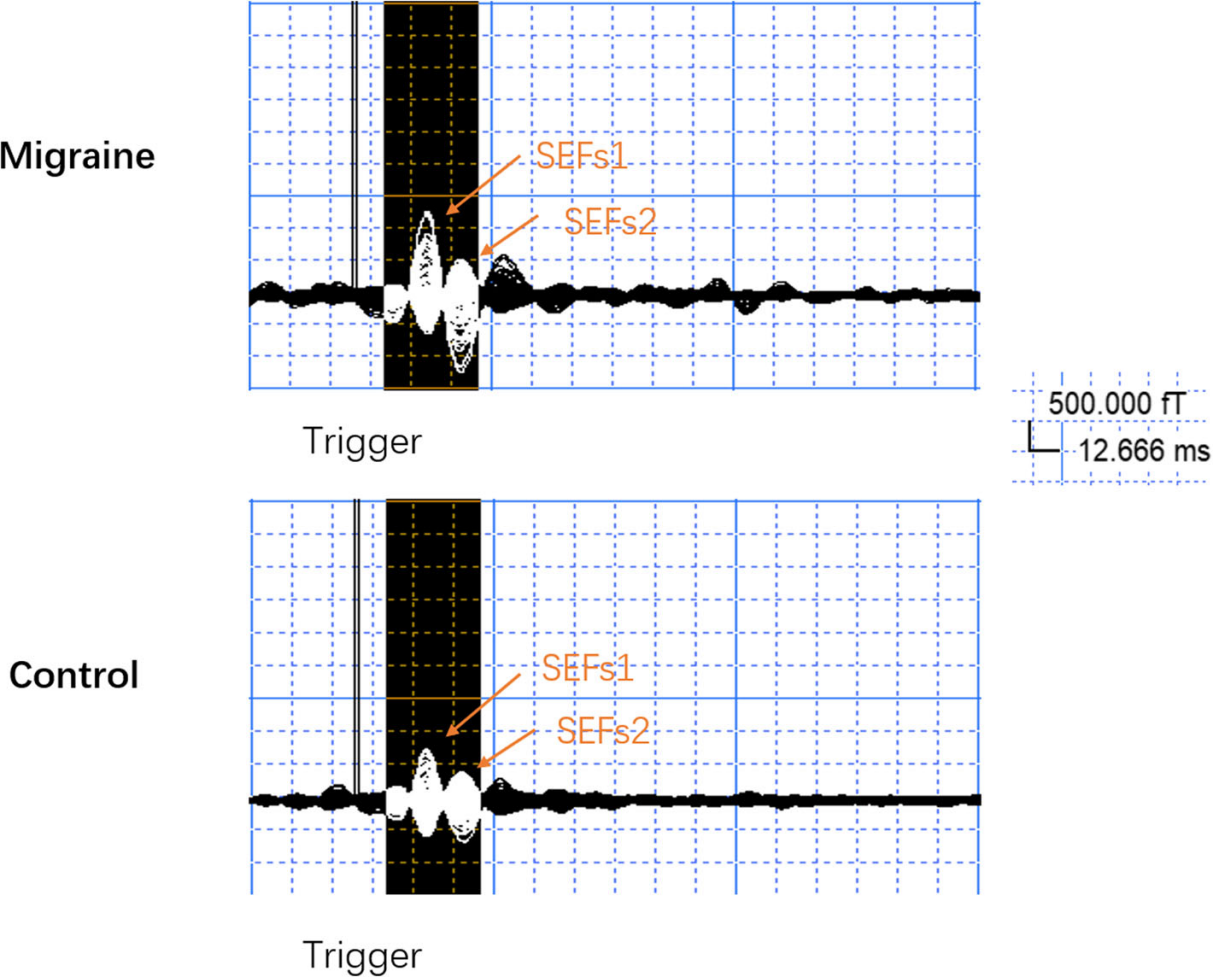
Magnetoencephalography (MEG) waveforms showing neuromagnetic activation evoked by right median nerve stimulation in a migraine subject (“Migraine”) and a healthy subject (“Control”) in a frequency band of 30–80 Hz. There are at least two responses in the somatosensory-evoked magnetic fields (SEFs), “SEFs1” and “SEFs2”. Our analyzed time window is 10-40 ms, which contained the two main responses under somatosensory stimulation. The “Trigger” indicates the start of median nerve stimuli. The “SEFs1” and “SEFs2” indicate the first and second SEF responses, respectively

The present study investigated somatosensory evoked high frequency signals, which were based on the average of 200 trials. This procedure keeps time-locked signals evoked by somatosensory stimulation while excluding artifacts or noise signals that are not “time-locked”. Furthermore, building on previous reports [24, 25], MEG waveforms were visually inspected by experienced clinicians to identify magnetic noise. The data were manually averaged after excluding the eye movement and muscular artifact. MEG data without noise or artifact were marked as “clean MEG data.” The averaged MEG data were preprocessed by removing the direct current (DC) offset based on the pre-trigger baseline, and the somatosensory-evoked magnetic fields were obtained. Neuromagnetic components (somatosensory-evoked magnetic responses) were identified in the averaged waveforms. Sample data are shown in Fig. 1.

#### Source localization

**Fig. 2:**
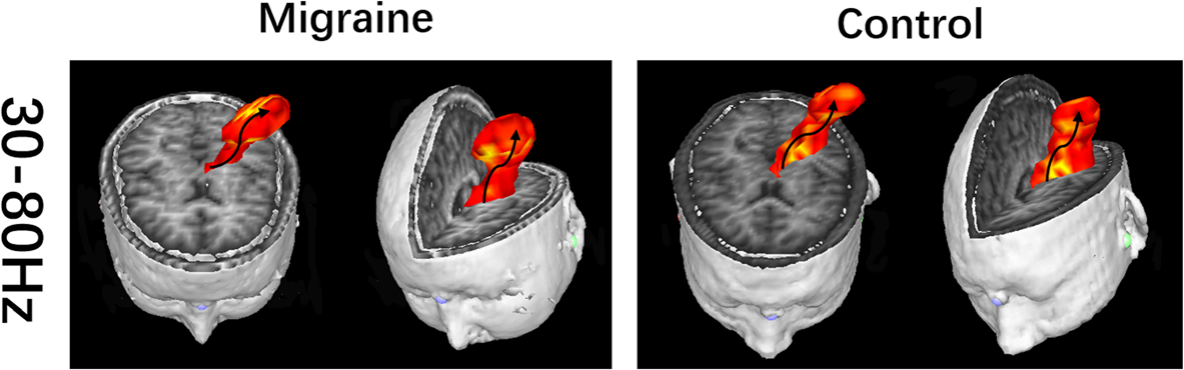
Real time source images showing spatial activities under somatosensory stimulation in gamma band (30–80 Hz) recorded from migraine patients and control. 3D images are displayed in axial, coronal and oblique sagittal positions. The black arrow indacates the source activity flow from the deep brain to the sensory cortex

To investigate the neuromagnetic network at source levels, we localized the significant neuromagnetic activity through real-time source imaging [26–29], which has been defined as the volumetric source activity over each time point and has been specifically developed and optimized to analyze activities in multiple frequency bands [28, 29]. We used two-step beamforming to calculate the source activity [30]. First, we computed lead fields for each source (or voxel position) and then generated matrices with the MEG data. Next, we selected sensors for partial sensor coverage for each voxel with a main field [30], which were named voxel-based partial sensors. The role of voxel-based partial sensors was to minimize the effect of coherent sources in source localization in the following beamform steps. The next step was to compute the covariance for the voxel-based partial sensors. Next, we computed two sets of magnetic source images using a vector beamformer [30]. Finally, the coherence source and source direction were estimated by using the covariance matrix vector beamformer. As soon as the source direction was confirmed, the final step was to generate the source activity (or virtual sensor waveform) through a scalar beamformer. Recent studies have described the algorithms and verifications in detail [26, 30]. In this study, the entire brain was scanned at a resolution of 6 mm (approximately 17,160 voxels/source). A sample of the data is shown in Fig. 2.

#### Estimating networks

Building on previous reports [30], networks were analyzed at the source level. To analyze FC at the source level, virtual sensor waveforms were computed for each source using the aforementioned algorithms. The neuromagnetic networks were estimated by analyzing the correlation of all virtual sensor signals in time-windows that corresponded to the somatosensory-evoked magnetic fields [30, 31]. In particular, the correlation of virtual sensor signals of two source pairs was statistically analyzed by calculating the correlation factor (or correlation coefficient). The correlation factors were calculated by the following equation:

1 $$ R\left({\mathrm{x}}_a,{\mathrm{x}}_b\right)=\frac{C\left({x}_a,{x}_b\right)}{S{x}_a{Sx}_b} $$

In this equation, *R*(*x*_*a*_, *x*_*b*_) indicates the correlation of a source pair in two locations (“a” and “b”). x_a_ and x_b_ indicate signals from two sources that were paired for computing connection. C (x_a_, x_b_) represents the mean of the signals in the two sources, while Sx_a_ and Sx_b_ represent the standard deviation of the signals from the two sources. Meanwhile, we analyzed every possible connection for each dual source pair at the source level to reduce possible bias.

The topology of the FC for each possible pair of voxel-based virtual sensors was co-registered to specific subject MRIs [26, 30]. Brain functional networks based on magnetic source imaging were visualized in three views (axial, coronal, and sagittal, respectively) to analyze the source connections; red was used to imply excitatory connections. (Fig. 4). Building on previous publications [32], an excitatory connection is a positive connection where the amplitude of signals in two connected sources are positively correlated. An inhibitory connection is a negative connection where the amplitude of signals in a source pair is negatively correlated. We used a threshold as a checkpoint to ensure the data quality. To determine the thresholding of connections, t values were computed for all source pairs.

2 $$ \mathrm{Tp}=\mathrm{R}\sqrt{\frac{K-2}{1-{R}^2}} $$

In eq. (2), Tp represents the t value of a correlation; R indicates the correlation of a source pair; K indicates the number of data points for connection. The T*p* value used had a corresponding *p* value < 0.01 as the thresholding for obtaining the FC network. The above algorithms were performed using the MEG Processor software (Cincinnati, OH, USA).

### Statistical analyses

The network pattern and odds ratio between patients and controls were visually inspected and analyzed by Fisher’s exact test. The two-tailed Student’s t-test was applied to assess the network parameters (degree, strength, path length and clustering coefficient) between the migraine and control groups. The correlations between migraine clinical characteristics (age, headache history, duration, frequency, VAS, and MIDAS) and MEG measurement (topographic patterns of the neural network and network parameters) were analyzed using Spearman’s correlation coefficients. The threshold of statistical significance for differences was set at *p* < 0.05 for each test. Considering the multiple comparisons, the significance level for each test was reduced from 0.05 to 0.00179 (four parameters×seven frequency bands, Bonferroni correction). A controlling procedure named false discovery rate (FDR) was widely applied to reduce type I errors [33]. Statistical analyses were implemented using the software package SPSS version 19.0 (IBM, Inc.)

## Results

### Clinical characteristics

This study included a total of twenty-two patients diagnosed with migraine without aura (29.27 ± 9.80 years; 15 females) and 22 controls (28.14 ± 7.11 years; 15 females), who were age- and sex-matched. Of these migraine patients, 8 presented bilateral headaches (36%), while 14 presented unilateral headaches (64%). The details are shown in Table 1.

**Table 1:** Clinical features and neuropsychological evaluation of patients

Parameter	Migraine	Control
Sex	7 M/15 F	7 M/15 F
Age (years)	29.27 ± 9.80	28.14 ± 7.11
History (years)	12.70 ± 7.32	
Frequency (times/month)	5.03 ± 3.78	
Durations of migraine attacks (hours)	23.80 ± 22.39	
Accompanied symptoms with attack N		
Phonophobia	19	
Photophobia	17	
Nausea/Vomiting	14	
Locus of headache N		
Bilateral	8	
Unilateral	14	
VAS (1–10)	7.82 ± 0.91	
MIDAS	52.18 ± 47.93	

*N* number, *VAS* visual analog scale, *MIDAS* the migraine disability assessment questionnaire

### Network pattern

**Fig. 3:**
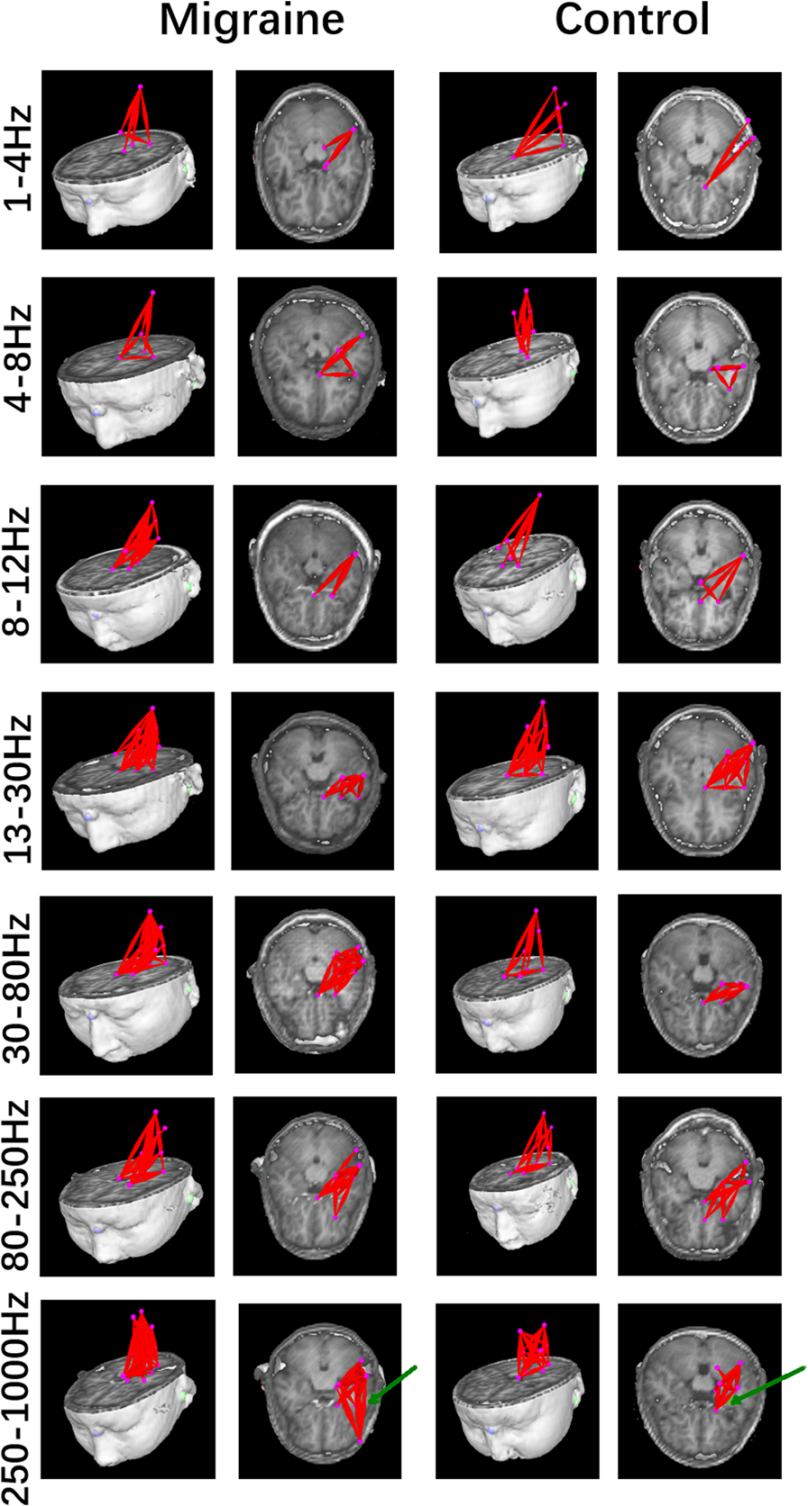
Typical functional connectivity network patterns in the 1–1000 Hz frequency range in migraine patients and controls, visualized from the lateral (left column) and axial (right column) views. Migraineurs show a significantly altered pattern of functional connectivity network at 250–1000 Hz compared with the controls, showing more connections between the sensory cortex and the frontal lobe. (Color figure online)

We found that in some frequency bands (1–4 Hz, 4–8 Hz, 8–12 Hz, 13–30 Hz, 30–80 Hz, and 80–250 Hz), both patients and controls showed strong excitatory connections between the thalamus and the ipsilateral primary sensory cortex, and there were also some connections among deep brain regions. The typical topographic distributions of functional connectivity patterns are shown in Fig. 3.

**Fig. 4:**
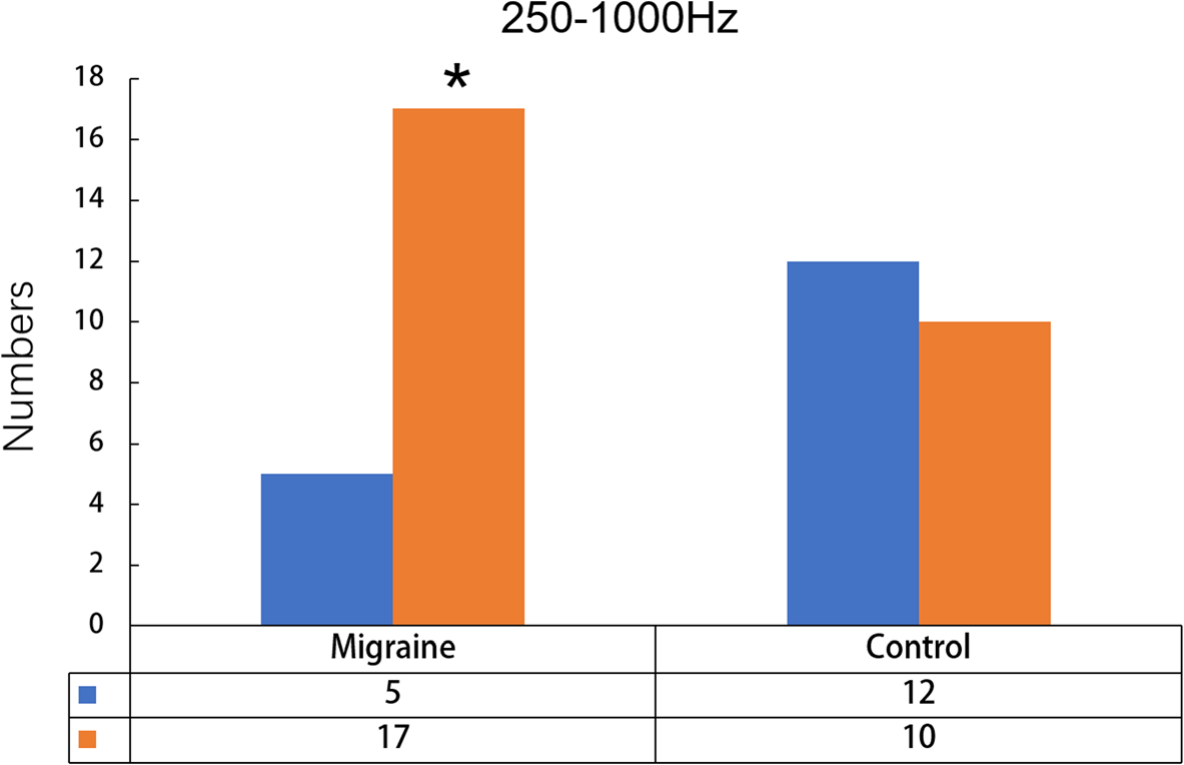
Number of migraineurs and controls with different functional connectivity patterns at 250–1000 Hz. Patients have significantly higher odds of functional connectivity in the frontal lobe than the controls. The orange bars indicate that functional connections are present in frontal cortices. The blues bars indicate that no excitatory connections exist in frontal cortices

In the high-frequency band (250–1000 Hz), a difference was found between patients and controls. In addition to the network between the deep brain area and the sensory cortex, most migraineurs (17/22) showed excitatory connectivity between the primary sensory cortex and the ipsilateral frontal cortex (Fig. 4). Details are shown in Fig. 3.

### Graph theory

#### Degree and strength

**Fig. 5:**
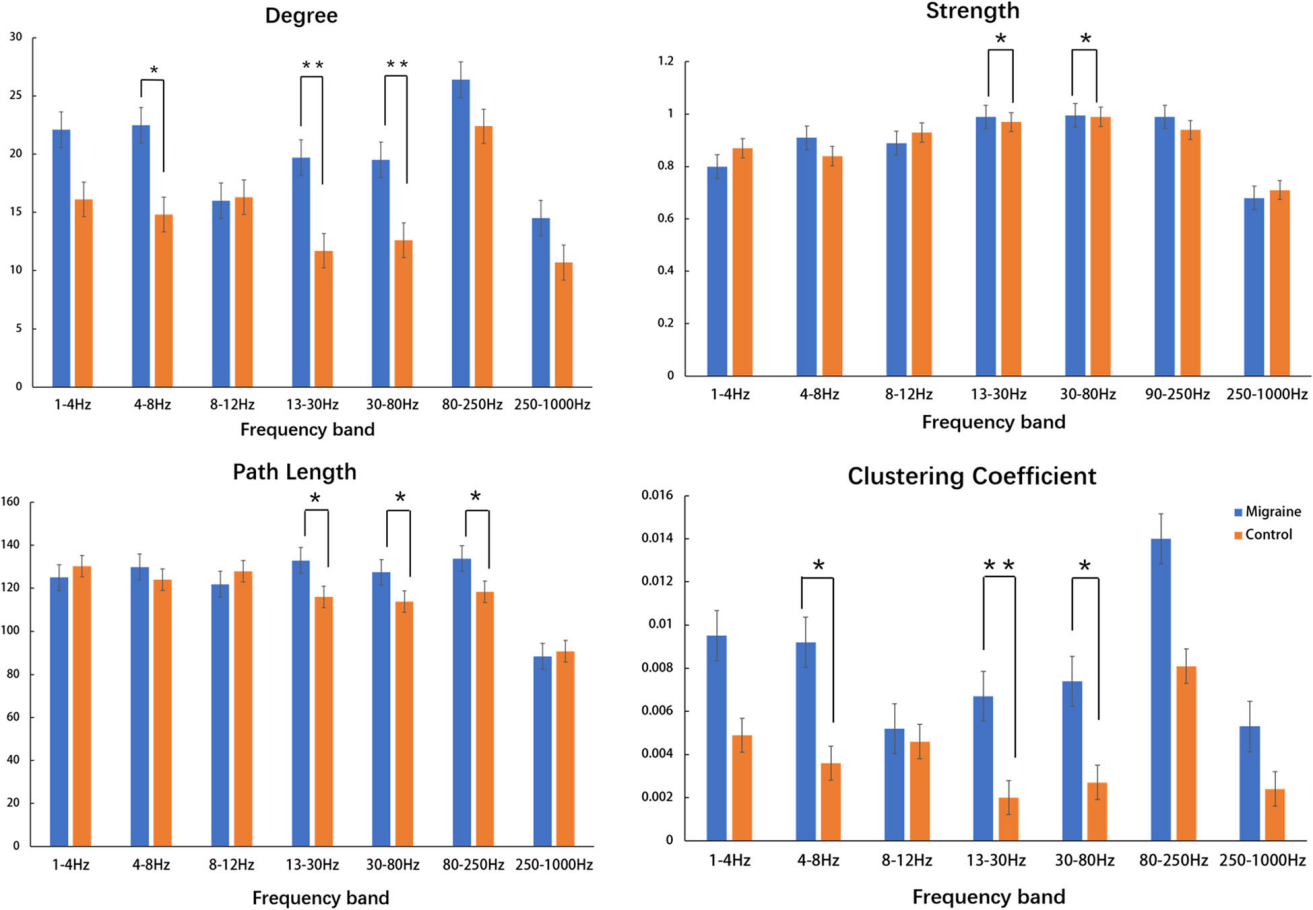
Comparison of the organization of the functional connectivity networks measured by four parameters (degree, strength, path length, and clustering coefficient) between migraine patients and healthy controls. *p < 0.05, and the result is still significant after correction for multiple comparisons using the FDR controlling procedure (corrected for 7 × 4 tests), **p < 0.01. (Color figure online)

Group comparisons revealed that the degree and strength of the functional connectivity network in the migraine patients were greater in some frequency bands compared to those of controls. The degree increased in the theta (4–8 Hz), beta (13–30 Hz), and gamma (30–80 Hz) bands. The strength increased in the beta (13–30 Hz) and gamma (30–80 Hz) bands. No significant difference was observed in other frequency bands. The degree and strength of seven frequency bands in both the migraine patients and the controls are shown in Fig. 5.

#### Path length and clustering coefficient

Group comparisons showed that the path length and clustering coefficient of the FC network in migraineurs increased in some frequency bands. The path length increased in the beta (13–30 Hz), gamma (30–80 Hz) and ripple (80–250 Hz) bands. The clustering coefficient increased in the theta (4–8 Hz), beta (13–30 Hz), and gamma (30–80 Hz) bands. The path lengths and clustering coefficients in migraine patients and controls are shown in Fig. 5.

### Clinical association

**Fig. 6:**
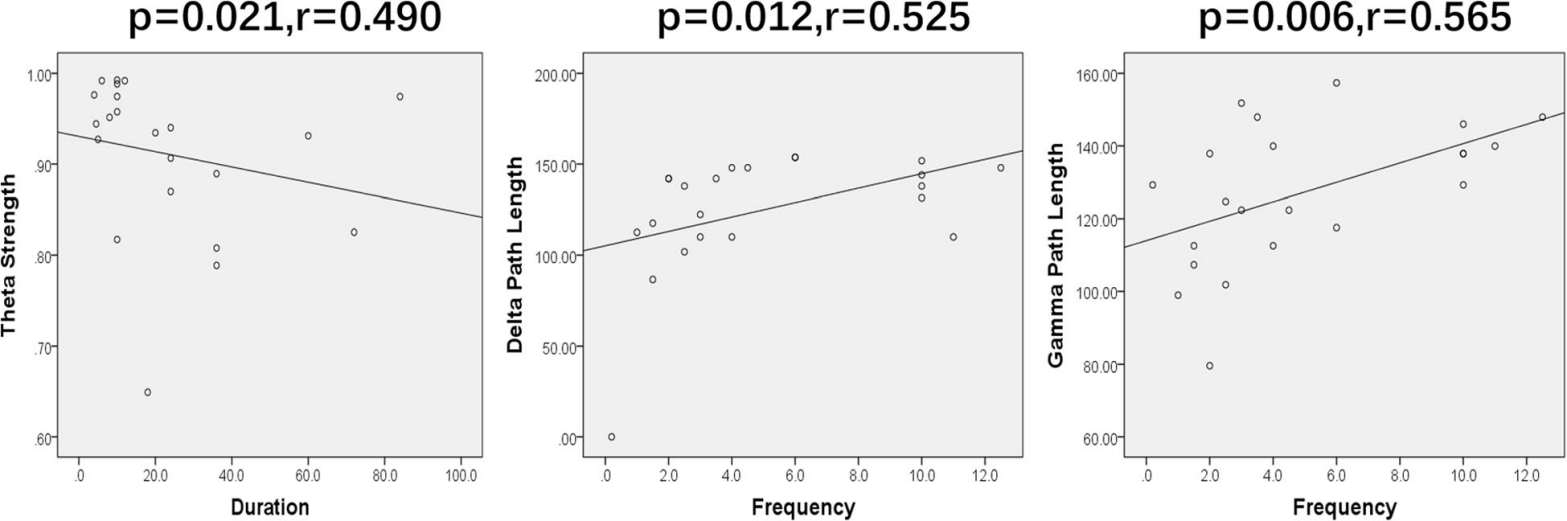
Charts of Spearman’s correlation showing significant relationships between the parameters of network and clinical characteristics in migraineurs. Duration of migraine attacks shows a negative correlation with network strength in the 4–8 Hz range. Migraine attack frequency shows a positive correlation with path length in both the 1–4 Hz and 30–80 Hz frequencies

The correlation analysis demonstrated no significant correlations between the clinical features (age, headache history, attack frequency, duration, VAS, and MIDAS) of the migraine patients and the topographic patterns of their brain network (*p* > 0.05). In seven frequency bands, there was no statistically significant association between the migraineurs’ age and four parameters in graph theory. We found that the headache frequency had a positive correlation with path lengths in both the delta band (*p* = 0.012, *r* = 0.525) and the gamma band (*p* = 0.006, *r* = 0.565). A significant positive correlation was found between the headache duration and strength in the theta band (*p* = 0.021, *r* = 0.490). Details are shown in Fig. 6.

## Discussion

In this study, we found frontal-lobe activation in migraine patients during median-nerve stimulation in the high-frequency band. The distribution of functional connectivity and graph-theory analysis indicated the differences in neural networks between the migraine and control group in some frequency bands.

The network pattern revealed an atypical connectivity under non-nociceptive electric stimulus in the migraine patients. Abnormal processing of sensory information in patients with migraine has been confirmed in other studies [17, 34]. Several sensory-discriminative brain regions in patients presenting migraine without aura tend to connect with each other into a firmly interconnected community [35]. Our results suggest that migraineurs exhibit different connective patterns between the sensory cortices and the frontal lobe during exposure to somatosensory stimuli. A previous EEG study demonstrated that patients with migraine showed different patterns of cortical activation after pain stimulation [15]. The functional connectivity of migraineurs in a resting state has changed for most researchers. Interestingly, an article from Denmark demonstrated no difference in neural connectivity between migraine sufferers and healthy subjects during the interictal phase. Several researchers hypothesized that the brain of patients with migraine with aura in the headache-free phase may dysfunction only when exposed to external stimuli [36]. Our finding of an abnormal network pattern in response to median-nerve stimulation indirectly supports this hypothesis.

The topological results also revealed that migraineurs have abnormal cortical activation between migraine attacks. Compared to the controls, the excess activation of the frontal lobe may be the result of cortical hyperexcitability [37, 38]. In our study, activation of the frontal lobe strongly suggests that the frontal cortex plays a key role in the pathology of migraine. We should mention that frontal regions are always activated by electrical stimuli, as for the late n30 wave [39],which were included in our analysis time-window. Based on whole-brain analysis, migraine patients show stronger activation in frontal cortex. The frontal lobe is mainly responsible for several psychological processes, including motor function, cognitive control, emotion, and social cognition [40]; additionally, the frontal cortex has been associated with pain control [41]. D’ Andrea proposed that cephalalgia attacks originate from an impairment in the top-down pathway that initiates in the frontal cortex in a highly excited brain, which subsequently leads to abnormal activation in the nuclei of the pain matrix [42]. Several lines of MEG studies have concluded that the frontal lobe exhibits high excitability in migraine patients [43, 44]. The role of the frontal lobe is the basis for the development of new therapeutic methods, unlike traditional medications, for migraine. Transcranial magnetic stimulation (TMS), in particular, repetitive TMS has been used to conduct noninvasive stimulation to the cortical areas. An rTMS study proved that such stimulus over the medial frontal lobe can suppress the central processing of pain perception [45]. Brighina and coworkers have confirmed that high-frequency rTMS in the left dorsal-lateral prefrontal cortex ameliorates chronic migraine [46].

Graph theory has revealed that the functional networks of migraineurs are notably different from those of controls. Small-World models are characterized by high clustering and short path length; therefore, they are widely used in neural networks, which confers the ability for specific information processing in a modular way among neighboring regions even over the whole network [47]. Deviation of the functional network topology in migraineurs has been demonstrated by other studies [48, 49]. In the present study, the abnormal networks can be characterized as increased degree in the theta, beta, and gamma bands; increased path length in the beta, gamma, and ripple bands; increased strength in the beta and gamma bands; and finally, an increased clustering coefficient in the theta, beta, and gamma bands. These findings suggest that the topological distribution in the functional networks of migraine patients deviated from the optimal. The degree is an often-used quantifier of centrality, and the strength is described as the sum of all neighboring link weights [50]. Increased degree and strength indicate a highly centralized anatomical network. The finding from Watts and his colleagues suggested that regular networks are characterized by not only a high clustering coefficient but also a very high path length [51]. The differences found in our study indicate that the functional network of migraine patients is more similar to a regular network than that of nonmigraine patients, which indicates an unbalanced functional integration and segregation. This finding demonstrates the abnormal network in sensory-related regions of the brain between migraine attacks.

Our results from the correlation analysis showed an association between the connectivity strength and duration of migraine in the theta band. Meanwhile, the path length in both the delta and gamma frequencies showed a positive correlation with the headache-attack frequency. An fMRI study has also correlated the duration of migraine attacks with abnormal networks [52]. On the other hand, structural MRI studies have demonstrated that the grey-matter volume decreases in some brain regions are related to estimated clinical parameters [53]. In summary, the correlation results in our study may result from both functional and structural changes.

We have recognized that this study has some limitations. Because we only recruited participants whose dominant hand is right, the lateralization of migraine may affect our result. Not all patients in the current study experienced bilateral headache; some of them experienced lateralization headaches. Further studies that stimulate both hands in migraineurs with lateralized headaches are needed to solve the above problems. The number of participants in our study is another limitation. More subjects would be needed in future research.

## Conclusions

This study demonstrates that migraine patients display altered functional connectivity in response to external stimuli during headache-free phases. Excess activation was observed in the cerebral cortex in addition to the sensory cortex, supporting the hypothesis that migraine attacks are related to cortical hyperexcitability. The dysfunction in the network may be associated with the pathogenesis of migraine. This result may contribute to understanding migraine pathophysiology and providing convincing evidence for a spatially targeted migraine therapy.

## Abbreviations

EEG: Electroencephalogram
FC: Functional connectivity
FDR: False discovery rate
fMRI: Functional magnetic resonance imaging
HFOs: High-frequency oscillations
ICHD: International Classification of Headache Disorders
MEG: Magnetoencephalography
MIDAS: The migraine disability assessment questionnaire
rTMS: Repetitive Transcranial magnetic stimulation
SEFs: Somatosensory-evoked magnetic fields
VAS: Visual analog scale
